# Coding Variants Coupled With Rapid Modeling in Zebrafish Implicate Dynein Genes, *dnaaf1* and *zmynd10*, as Adolescent Idiopathic Scoliosis Candidate Genes

**DOI:** 10.3389/fcell.2020.582255

**Published:** 2020-11-04

**Authors:** Yunjia Wang, Zhenhao Liu, Guanteng Yang, Qile Gao, Lige Xiao, Jiong Li, Chaofeng Guo, Benjamin R. Troutwine, Ryan S. Gray, Lu Xie, Hongqi Zhang

**Affiliations:** ^1^Department of Spine Surgery and Orthopaedics, Xiangya Hospital, Central South University, Changsha, China; ^2^National Clinical Research Center for Geriatric Disorders, Xiangya Hospital, Central South University, Changsha, China; ^3^Department of Pediatrics, Dell Pediatric Research Institute, The University of Texas at Austin, Dell Medical School, Austin, TX, United States; ^4^Shanghai Center for Bioinformation Technology, Shanghai Academy of Science and Technology, Shanghai, China; ^5^Key Laboratory of Carcinogenesis and Cancer Invasion, Ministry of Education, Key Laboratory of Carcinogenesis, National Health and Family Planning Commission, Xiangya Hospital, Central South University, Changsha, China

**Keywords:** adolescent idiopathic scoliosis, whole exome sequencing, southern Chinese population, genetic variations, bioinformatics analysis, gene knockout

## Abstract

Adolescent idiopathic scoliosis (AIS) is the most common pediatric spine disorder affecting ∼3% of children worldwide. Human genetic studies suggest a complex polygenic disease model for AIS with large genetic and phenotypic heterogeneity. However, the overall genetic etiology of AIS remains poorly understood. To identify additional AIS susceptibility loci, we performed whole-exome sequencing (WES) on a cohort of 195 Southern Chinese AIS patients. Bioinformatics analysis identified 237 novel rare variants associated with AIS, located in 232 new susceptibility loci. Enrichment analysis of these variants revealed 10 gene families associated with our AIS cohort. We screened these gene families by comparing our candidate gene list with IS candidate genes in the Human Phenotype Ontology (HPO) database and previous reported studies. Two candidate gene families, axonemal dynein and axonemal dynein assembly factors, were retained for their associations with ciliary architecture and function. The damaging effects of candidate variants in dynein genes *dnali1*, *dnah1*, *dnaaf*, and *zmynd10*, as well as in one fibrillin-related gene *tns1*, were functionally analyzed in zebrafish using targeted CRISPR/Cas9 screening. Knockout of two candidate genes, *dnaaf1* or *zmynd10*, recapitulated scoliosis in viable adult zebrafish. Altogether, our results suggest that the disruption of one or more dynein-associated factors may correlate with AIS susceptibility in the Southern Chinese population.

## Introduction

Adolescent idiopathic scoliosis (AIS) represented the majority of scoliotic disorders. The prevalence rate of AIS was approximately 1.0–5.1% among children (Zhang et al., 2015; Hengwei et al., 2016; Diebo et al., 2019). One of the hallmarks of AIS is the absence of obvious abnormalities of the vertebrae and without obvious neuromuscular dysfunction. Phenotyping of AIS is typically evaluated based on the curve bending magnitude, which is usually referred to as the Cobb angle. A subset of AIS patients displaying more severe, progressive curves require costly surgical treatment, with long-term health implications (Cheng et al., 2015). In addition to the angle of spine curvature, additional variables including major curve direction, curve shape, location of the apical vertebrae, and curve length have been proposed in order to facilitate a more comprehensive classification of the sub-phenotypes of AIS (Lenke et al., 2003).

The etiology of AIS remains poorly understood, yet several hypotheses including abnormalities in genetics, skeletal spinal development, bone metabolism, and other processes have been proposed (Burwell et al., 2016). It is widely reported that AIS has a strong genetic predisposition (Grauers et al., 2016). However, given the lack of readily identifiable monogenic traits associated with familial AIS, it is likely that AIS is a multifactorial disease with a range of tissue-level origins. The paucity of molecular genetic insight for AIS makes it difficult to perform early detection or to predict curve progression in the clinic. Recent efforts of genome-wide association study (GWAS) analyses and family based whole-exome sequencing (WES) studies of AIS have identified disease-candidate loci and disease-associated variants. Unbiased GWASs from diverse ethnic groups implicate several loci near a range of genes including *LBX1*, *GPR126*, *BNC2*, *PAX1*, and *AJAP1* (Takahashi et al., 2011; Kou et al., 2013; Sharma et al., 2015; Zhu et al., 2017). Genomic sequencing approaches have identified mutations in familial AIS pedigrees, including mutations in *HSPG2*, *POC5*, *AKAP2*, *MAPK7*, and *CELSR2* genes (Baschal et al., 2014; Patten et al., 2015; Li et al., 2016; Einarsdottir et al., 2017; Gao et al., 2017). WES in AIS cohort indicated that rare variants in *FBN1/2*, musculoskeletal collagen genes, including 14 collagen genes, and cilia-associated genes, were enriched in AIS patients (Buchan et al., 2014; Haller et al., 2016; Baschal et al., 2018). Together, these efforts suggest a complex polygenic disease model for AIS, encompassing a range of genetic and phenotypic heterogeneity. Altogether, the current variations reported thus far only explain approximately 5% of disease occurrence. For this reason, we set out to expand our understanding of genetic variants associated with AIS by a focused exome sequencing of Southern Chinese people.

The aim of this study was to detect rare variants associated with AIS susceptibility in a Southern Chinese population. WES data were obtained from 195 sporadic AIS patients. Using distribution comparison filtering and deleterious allele prediction, we identified several novel rare variants associated with AIS in independent loci. Five clinical characteristics of scoliosis were also assessed, including Cobb angle, curve shape, sideness of the major curve (e.g., left or right), the axial location of the apical vertebrae, and the number of tilted vertebrae. By comparing the different subgroups of AIS, we determined several severity-correlated genetic factors and phenotype–genotype associations. To functionally verify the role of candidate genes associated with AIS in our cohort, we assayed a group of related genes highly enriched in functional gene families involved in axonemal/cilia biology using CRISPR/Cas9 genome editing in zebrafish. This study may provide new insights to the molecular genetics of scoliosis in human AIS.

## Materials and Methods

### Patients and Clinical Characteristics

This study was approved by the Ethics Committee of Xiangya Hospital of Central South University (Changsha, China), and all included subjects and their legal guardians gave their written informed consent to participate in the study. A total of 195 unrelated sporadic AIS patients were recruited from Xiangya Hospital, which is one of the major centers for treating AIS patients in China. All AIS cases were from the ethnic Han population of Southern China. The inclusion criteria were as follows: 1) diagnosed with AIS by clinical and radiological findings, confirmed by three spine surgeons; 2) maximum Cobb angle >10°; and 3) all second-degree family members do not have scoliosis or inherited diseases. Patients with determined etiology, like congenital vertebral malformations, neuromuscular disorders, skeletal developmental delay, and connective tissue diseases, were excluded from the study.

### Whole-Exome Sequencing and Data Preprocessing

Genomic DNA was extracted from the venous peripheral blood of AIS patients. The qualified genomic DNA sample was randomly fragmented and ligated with adapters. The extracted DNA was amplified, purified, and hybridized to the exome array for enrichment. After non-hybridized fragments were washed out, captured products were used to estimate the magnitude of enrichment. Qualified captured library was then loaded onto BGISEQ-500 sequencing platforms for high-throughput sequencing. The sequence data of each individual were generated as paired-end reads, defined as “raw data” and stored in the FASTQ format. Raw data were filtered before progressing to bioinformatics analysis using the following criteria: 1) reads containing sequencing adapters were removed. 2) Reads with more than 50% low-quality base (base quality ≤ 20) were removed. 3) Reads with unknown base (“N” base) ratio of more than 10% were removed.

### Alignment and Variant Calling

All clean data for each sample were mapped to the human reference genome (hg19/GRCh37) with the Burrows-Wheeler Aligner (BWA). To ensure accurate variant calling, recommended Best Practices for variant calling with the Genome Analysis Toolkit^1^ (GATK, v3.3.0) were performed. Local realignment around InDels and base quality score recalibration was performed, with duplicate reads removed by Picard tools. The sequencing depth and coverage for each individual sample were further calculated. Genetic variations were then detected. Based on the above variant calling results, several criteria were used to filter for high-confidence variations: a) the variation sequencing depth should be ≥30; b) allele frequency (AF) of the variation in sequencing reads should be 0.4 < AF < 0.6 (heterozygous variation) or AF > 0.9 (homozygous variation); c) the single nucleotide variants (SNVs) must have only one type of altered nucleotide.

### Functional Annotation and Deleterious Variation Prediction

Annotations for variants were carried out with the SnpEff tool and ANNOVAR. For the above-mentioned filtered high-confidence variants, multiple *in silico* methods including SIFT, Polyphen2, LRT, FATHMM, and MutationTaster were performed for deleterious function prediction. An accurate prediction of deleterious variants is a key component of assessing their contribution to disease phenotype. Variations predicted as deleterious or probably damaging in two and more methods were treated as functional variations and retained for further analysis.

### Statistical Analysis and Adolescent Idiopathic Scoliosis-Associated Variant Identification

The high-confidence variants were analyzed for AIS association. For the variation distribution analysis, Fisher’s exact test and chi-squared test were performed with allele mutant count and mutant samples in different populations. AFs of variants contained in both AIS and control dataset [data from 105 samples of Southern Han Chinese (CHS) population from 1000 Genomes Project (1000G) and 4,327 samples of East Asian (EAS) population from ExAC] were compared between the two groups by the variation distribution analysis. In addition, variants contained in more than two samples in AIS dataset, with no frequency in 1000G or ExAC, were defined as novel variants associated with AIS. The Wilcoxon rank sum test was applied to identify variations that were associated with clinical characteristics. *P*-values were calculated with false discovery rate (FDR) correction using the Benjamini and Hochberg method. All the statistical analyses were performed with R software (Version, 3.5.2).

CHS population in 1000G and EAS population in ExAC database were used for normal variation distribution analysis of similar genetic background, to compare with variation distribution in our Southern Han AIS cohort. Variants with a higher frequency than 5% in both Exome Sequencing Project (ExAC) and 1000G were filtered away. Variants predicted with deleterious functions were kept. Further functional analyses including Gene Ontology (GO), Kyoto Encyclopedia of Genes and Genomes (KEGG) pathway and gene family enrichment analysis were performed for biological interpretation of these AIS-associated genetic variants. Gene family information was downloaded from HUGO Gene Nomenclature Committee (HGNC) database. Association of AIS clinical features and variants was also analyzed. Family genes interactions were analyzed by protein–protein interaction network.

### Variation Structural Visualization

MutationMapper in cBioPortal was used to generate a lollipop plot, which can map the variants onto linear protein sequence and its domains. Phyre^2^ (Kelley et al., 2015) was used to predict the secondary and tertiary protein structures of the variant-related gene, based on sequence homology alignment to PDB. Usually, the top template is selected as the structure model. ProQ2 was performed to assess the model quality, and HHsearch provided the alignment confidence. PSI-Blast Residue was applied to demonstrate amino acid preference and conservative property at the variant position of the protein. Further, the predicted 3D structure was visualized in Missense3D (Ittisoponpisan et al., 2019), and the structural change introduced by the variant amino acid substitution can be predicted and visualized on 3D dimension. A nucleotide variant that falls into functional domain on protein linear structure, or is highly preferred or conserved in its original form and position, or predicted to be deleterious, or causes amino acid change and structural alteration is usually considered as a functional variant and is qualified for further functional validation in an animal model.

### Zebrafish as Gene Function Phenotype Model

Zebrafish experiments were performed in accordance with approved Institutional Animal Care and Use Committee (IACUC) protocols. All procedures involving zebrafish were approved by the Animal Studies Committee at the University of Texas at Austin (AUP-2018-00342). Adult AB (wild-type) zebrafish were maintained and raised at the Zebrafish Aquatic Housing Systems (Aquaneering, San Diego, CA, United States) with 28.5°C and 14/10-h light–dark periodicity.

### CRISPR/Cas9 System Methods for Gene Disruption

For each locus, four single-guide RNAs (sgRNA) were synthesized using a common TRACR RNA template oligo. Then four gene-specific oligos (Supplementary Table S7) were synthesized for each locus using T7 RNA Polymerase (New England Biolabs, Ipswich, MA, United States). Last, each template pools for *in vitro* transcription reactions were purified using Zymo RNA Clean and Concentrator kit (Zymo, Irvine, CA, United States) as the protocol. For generating CRISPR/Cas9 ribonucleoprotein (Cas9/RNP) complexes prior to injection, 31 μM of sgRNA mixture was incubated with 5 μM of Cas9 protein (Integrated DNA Technologies, Coralville, IA, United States) for 5 min at room temperature. We injected ∼500 pl of Cas9/RNP mixture into the yolk of a one-cell stage zebrafish embryo. All the embryos for each independent gene target injection were obtained from a mass mating of AB wild-type fish to ensure genetic diversity. Embryos were raised in egg water (60 μg/ml of sea salts in distilled water) with methylene blue at 28.5°C. Postinjection screening was done at 1, 3, and 5 days postfertilization (dpf).

### RNA Isolation and Real-Time PCR

After Cas9 system injection, zebrafish with body curvatures phenotypes were screened and isolated at 3 dpf. Total RNA was isolated using TRIzol Reagent (Invitrogen, Carlsbad, CA, United States) according to the manufacturer’s protocol. In brief, 50 zebrafish embryos were collected and anesthetized in 0.16% tricaine/egg water solution and treated with 1 ml of TRIzol immediately. RNA was cleaned up with the Qiagen RNEasy Mini Kit (Qiagen, Germantown, MD, United States). cDNA was synthesized using iScript cDNA synthesis kit (Bio-Rad, Hercules, CA, United States). Real-time PCR (RT-PCR) was performed on a BioRAD CFX96 RT PCR detection system, using a SYBR Green Supermix (Bio-Rad, Hercules, CA, United States). Gene expression was normalized to *elongation factor 1 alpha* (*elfa*) mRNA. The relative expression levels of *dnaaf1* and *zmynd10* were calculated using the 2^–(ΔΔ*Ct*)^ method. Specific primers sequences were designed as follows: *dnaaf1* forward, 5′-TCCACATCGATGAACGCGG, and reverse, 5′-AGTCTTTGGTAATTCTGGGGC; *zmynd10* forward, 5′-GTG GGACGCTCTGTATCTGG, and reverse, 5′-ACTTTGCTTTTG CAGATCCTGT; *elfa* forward, 5′-CTTCTCAGGCTGACTGT GC, and reverse, 5′-CCGCTAGCATTACCCTCC.

### Skeletal Staining and Imaging for Zebrafish Spine Development Phenotyping

Tricaine anesthetized adult fish were fixed in 10% formalin overnight, incubated in 100% acetone overnight, and stained with an acidic Alcian Blue/Alizarin Red ethanol solution at 37°C overnight. Clearing was performed with 1% KOH with multiple changes of solution. After the clearing, specimens were further cleared sequentially with increasing glycerol concentrations up to 80% (diluted in 1% KOH). Bright-field imaging of zebrafish and skeletal stained specimen was taken using an Olympus SZX2-ILLT microscope (Olympus, Tokyo, Japan).

## Results

### Patients and Exome Sequencing Data Overview

A total of 195 AIS patients were included in this project, and detailed clinical characteristics of the patient cohort are shown (Supplementary Table S1). Detailed description of AIS subjects showed that the average age at diagnosis of the patients was 14.93 ± 3.04 years old, and the mean Cobb angle was 41.88 ± 18.35°. There were 39 male cases and 156 female cases, and the female–male ratio was 4. In addition, five clinical manifestations were measured to evaluate the states of AIS patients, including curve shape, left or right main curve, location of the apical vertebra, and number of tilted vertebrae. The curve pattern was defined as either a “C” or “S” shape; 131 “C”-shaped curves and 64 “S”-shaped curves were included. Fifty-two left main curves and 143 right main curves were included. According to the position of the apical vertebra, two categories were classified: 106 patients with apex located between T1 and T9 vertebrae and 89 patients with apex under T10 vertebra. With the tilted vertebrae construct of the whole curve, the number was counted from the vertebra of which the body began to tilt to when body tilt disappeared in the coronal plane, and long or short curves (longer or shorter than mean value) may be defined. The mean number was 13.00 ± 3.84 in our cohort.

To improve the understanding of genetic variants in AIS susceptibility, WES was performed for this AIS cohort. On average, 23.35 × 10^11^ bases (range, 12.67 × 10^11^–34.81 × 10^11^) per sample with a mean coverage of 99.88% (range, 99.82–99.98%) and a mean sequencing depth of 257 (range, 134–380) were detected; and average of 99.63% (range, 98.54–99.86%) of the targeted exome sequences were covered by more than 10 reads, exhibiting good sequencing quality and coverage. Variant calling identified 1.02 × 10^6^ single-nucleotide polymorphisms (SNPs) (range, 8.96 × 10^5^–1.18 × 10^6^) per sample, including 1.75 × 10^5^ (range, 1.45 × 10^5^–2.22 × 10^5^) insertions or deletions (indels) per sample and 1.08 × 10^6^ coding SNPs in total. Eventually, about 1.38 × 10^6^ SNPs with the sequencing depth ≥30 passed data preprocessing and were included in follow-up bioinformatics and statistical analysis, which were of acceptable WES data discovery scale.

### Identification of Adolescent Idiopathic Scoliosis-Associated Variants and Functional Analyses

**FIGURE 1:**
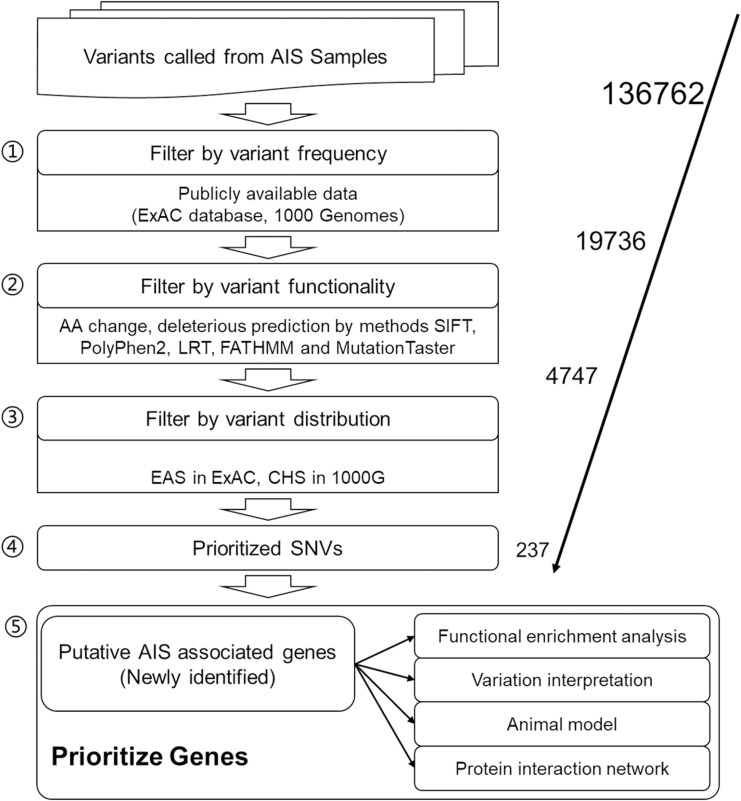
Workflow of adolescent idiopathic scoliosis (AIS) associated variant study.

After data preprocessing, 136,762 SNVs with the sequencing depth ≥30 were selected as high-quality called variants for further analyses. For result repeatability, only variants mutated in two or more patients were kept, and SNVs with mutated AF of more than 5% in both ExAC EAS population and 1000 Genomes CHS population were filtered away because they are considered general polymorphism in the population. These two steps rendered 19,736 SNVs remaining for further filtering. Five *in silico* function deleterious prediction methods were performed, and 4,747 SNVs were found with deleterious functions in two and more methods. Further, Fisher exact test was applied to identify SNVs differentially distributed between AIS population and ExAC EAS population or 1000 Genomes CHS population. Finally, 237 SNVs that are novel variants (not existing in the database) or significantly distributed in AIS cohort (in the database but the distribution rate is statistically different from that of AIS) were obtained as AIS-associated variants in this study (Figure 1; Supplementary Table S2), and they correspond to 232 genes.

**FIGURE 2:**
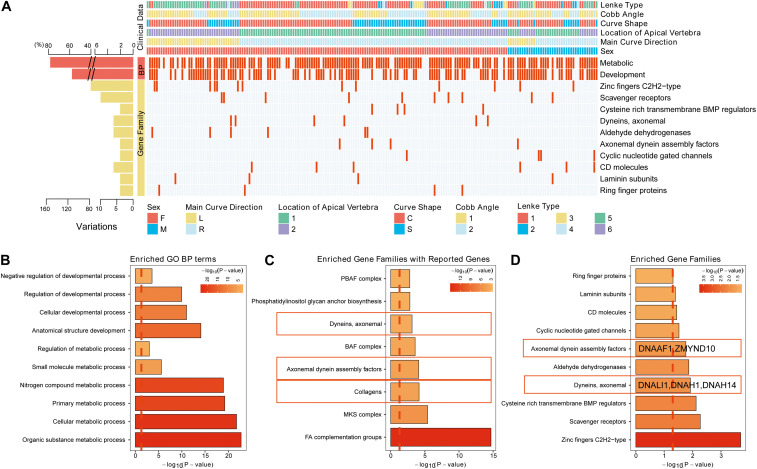
The variants identified from Southern Chinese AIS patients. **(A)** Genetic variations detected in gene families and related biological processes (BPs). Each row represents an individual gene family or BP category. The altered individuals and frequency of each gene are noted on the left. Each column representing an affected sample with clinical features is indicated by the color key. The occurrence of a variant with gene in corresponding terms across an individual sample is shown by a red (missense variant) or blue (no variant) color scheme. Sex, F = female, M = male. Main curve direction represents the sideness of the major curve, L = left, R = right. Location of the apical vertebra represents the axial location of the apical vertebrae, 1 = T1–T9 vertebrae, 2 = under the T10 vertebra. Curve shape, C = “C”-shaped curve, S = “S”-shaped curves. Cobb angle, 1 = below the mean Cobb angle, 2 = above the mean Cobb angle. Lenke type, 1 = type 1, 2 = type 2, 3 = type 3, 4 = type 4, 5 = type 5, and 6 = type 6. **(B)** The top 10 enriched Gene Ontology (GO) BP terms in our study. **(C)** The top enriched gene families with the genes in previous studies or database. **(D)** The top enriched gene families with the genes reported in our study.

To further explore the critical gene sets and pathways in AIS susceptibility, gProfileR and gene set enrichment analysis (GSEA) were performed on the 232 genes. The enriched GO biological process (BP) terms could be divided into two categories, metabolic and developmental processes. The variants, distributing irregularly, of metabolic, developmental processes and enriched gene families showed no significant convergence with disease subgroups as depicted in the diagram (Figure 2A). However, the 10 most enriched GO BP terms were related to bone development, cellular process, and metabolism, such as organic substance metabolic process and biosynthetic process (Figure 2B). Many disease-related genes are paralogs or belong to similar gene families (Yandell et al., 2008). Using a polygenic model of disease, we first investigated the enriched gene families of genes associated with AIS reported in previous articles or databases (Figure 2C). We noticed in SNVs among axonemal dynein, axonemal dynein assembly factors as these components are required for cilia physiology, which is associated with scoliosis phenotypes in multiple mutant zebrafish models (Bachmann-Gagescu et al., 2011; Grimes et al., 2016; Konjikusic et al., 2018; Van Gennip et al., 2018). Then we investigated the distribution of the candidate gene families among our filtered variants (Supplementary Table S3). From these 10 gene families found to be enriched in our AIS cohort based on the hypergeometric distribution and ranked by -Log10 (FDR) value, two gene families, axonemal dynein and axonemal dynein assembly factors, overlapped with previous discovery displayed in Figure 2C (Figure 2D). These two gene families are associated with cilia structure and function and contain five genes in total: *DNALI1*, *DNAH1*, and *DNAH14* from axonemal dynein family, and *DNAAF1* and *ZMYND10* from axonemal dynein assembly factors. Repeated results from reported AIS associated genes and our candidate genes suggest that variants in axonemal dynein assembly factors and axonemal dynein genes may play an important role in AIS pathogenesis.

### Interpretation of Variants in Dynein-Associated Genes

**FIGURE 3:**
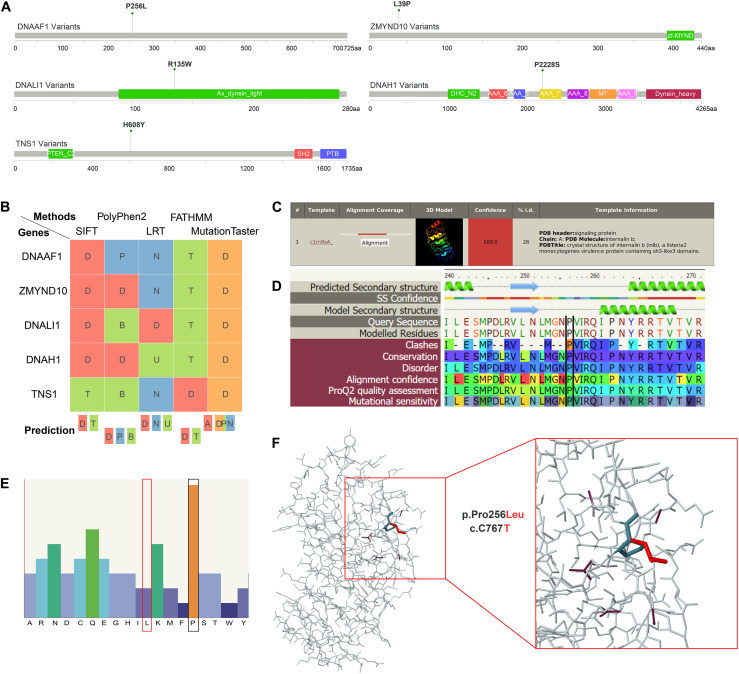
Structural interpretation of genetic alteration in putative adolescent idiopathic scoliosis (AIS)-related genes. **(A)** Lollipop plot for mapping mutations on the linear protein and its domains. P256L is the significant amino acid change caused by single nucleotide variants (SNVs) on DNAAF1. **(B)** Deleterious prediction by *in silico* methods. D means deleterious in SIFT, LRT, and FATHMM. In PolyPhen2, D means probably damaging. And in MutationTaster, D means disease causing. **(C)** The detailed template information and models of DNAAF1. The top template was selected and analyzed in the downstream analyses. **(D)** The sequence view displays the predicted secondary structure of DNAAF1, the confidence in this prediction, the secondary structure of the model, the sequence, and the modeled regions. The corresponding result scores for model assessment are shown with colors. **(E)** The sequence profile graph of DNAAF1 represents residue preferences at a particular sequence position. **(F)** The predicted 3D structure of the mapping domain on DNAAF1. Wild type (dark color) and mutant type (red color) of the variant are shown.

Although dynein family genes related to axonemal structure organization and motility were reported to be associated with AIS before (Baschal et al., 2018), the variants of four out of the above five genes identified in our WES study of Southern Chinese patients are novel. These variants are located on *DNALI1* and *DNAH1* genes from axonemal dynein family, and *DNAAF1* and *ZMYND10* genes from axonemal dynein assembly factors. For comparison, we included another non-dynein family gene *TNS1* (Tensin 1) with novel variant discovered in our WES data for further investigation. *TNS1* is involved in fibrillar adhesion formation, cell migration, cartilage development, and linking signal transduction pathways to the cytoskeleton. We used a systematic approach, including protein structural alteration prediction as well as several pathogenicity prediction methods, to elucidate the damaging effect of these five candidate variants in cytoskeleton movement associated genes before *in vivo* validation. First, conserved domain of candidate variants and correspondent amino acid change were depicted (Figure 3A). Second, positive deleterious function prediction results of these variants were shown (Figure 3B). Third, secondary structure alteration caused by these variants and the amino acid preference of each residue at the location of these variants were presented (Figures 3C,D and Supplementary Figures S1–S4). Finally, the predicted 3D structure alterations of available mapping domains were determined. To illustrate one example, for *DNAAF1* gene, one variant (rs764943936) was found to be significantly associated with AIS (Figure 3A). We selected a 3D structure with 100.0 confidence from phyre2 (Figure 3C) for structure alteration analyses. Predicted secondary structure and results of the following analyses (conservation, disorder prediction, etc.) of the single amino acid alternation (P256L) were shown (Figure 3D). The top amino acid preference of this position is proline, while substitution by leucine scored much lower (Figure 3E), suggesting that this P256L substitution may be functionally damaging DNAAF1 protein function. The overall 3D structure was depicted showing specific position structure change caused by proline to leucine substitution (Figure 3F).

### Dynein-Associated Genes Result in Body Curvature and Scoliosis Phenotypes in Zebrafish

**FIGURE 4:**
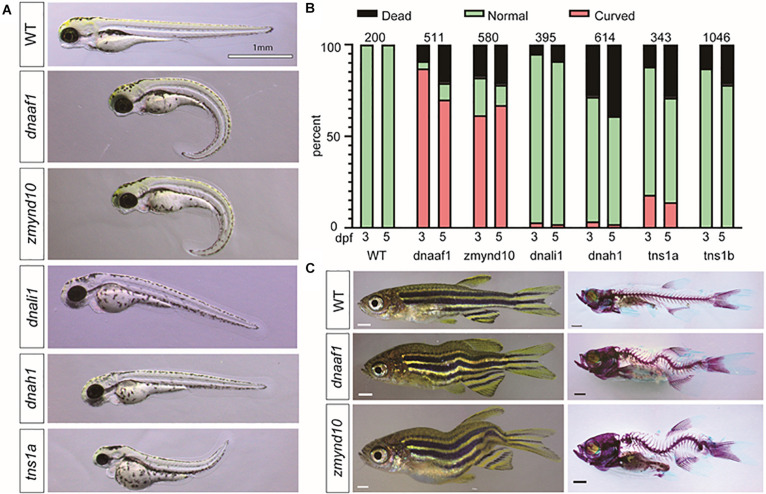
CRISPR/Cas9 knockout of candidate genes in zebrafish recapitulate the scoliosis phenotype. **(A)** Representative phenotypes of zebrafish larvae 3 dpf (days postfertilization) that were injected with multiplexed CRISPR/Cas9 reagents to target mutagenesis to candidate adolescent idiopathic scoliosis (AIS) associated genes. **(B)** Histograms representing the total percentage of curve body phenotype assessed at 3 and 5 dpf after injection is shown in the bar graph with red color. The number of larval fish assessed for each targeted gene is displayed in each set of bars. **(C)** Bright-field images (left) and Alizarin red/Alcian Blue stained (right) zebrafish (>30 dpf). All scale bars: 1 mm.

Faithful genetic models of scoliosis, which model aspects of AIS in zebrafish, have been described (Kusumi and Dunwoodie, 2010; Boswell and Ciruna, 2017; Wu et al., 2019). Thus far, the etiology of scoliosis in these zebrafish models is associated with defects in the development of ependymal cell cilia and abnormal cerebrospinal fluid (CSF) physiology (Grimes et al., 2016; Konjikusic et al., 2018), disassembly of the central canal resident glycoprotein structure called the Reissner fiber (Lu et al., 2020; Rose et al., 2020; Troutwine et al., 2020), and alteration of neuropeptide signaling within the central canal (Zhang et al., 2018; Vesque et al., 2019; Lu et al., 2020), which altogether appear to contribute to increased neuroinflammation (Van Gennip et al., 2018; Rose et al., 2020). Given the increased incidence of variants in axonemal dynein assembly factors and axonemal dynein genes in our cohort, which are well established to be essential for cilia physiology, we set next out to test if these genes were essential for spine morphogenesis in zebrafish. To rapidly test these candidate genes, we used a robust F0 CRISPR/Cas9 screening approach, which utilizes four guide RNAs targeting multiple regions of the same genetic loci (Wu et al., 2018). Using this approach, we screened six independent loci, corresponding to five dynein-associated genes interpreted before, which encompass all known paralogs of these genes in zebrafish (Supplementary Table S8). In total, five targeted genes lead to body curvature phenotypes during early larval development (Figure 4A), and of these two, targeted genes lead to adult-viable scoliosis phenotypes in adult zebrafish after CRISPR/Cas9 targeted mutagenesis (Figure 4B). Targeted disruption of the *dynein*, *axonemal*, *assembly factor 1* (*dnaaf1*) (previously *lrrc50*), and *zinc finger*, *MYND-type containing 10* (*zmynd10*) genes both demonstrated a consistently high incidence of severe body curvature phenotypes (Figure 4C and Supplementary Table S8), which is consistent with stable mutant phenotypes reported for these genes previously (Sullivan-Brown et al., 2008; Zhang et al., 2018). The majority of these *dnaaf1* or *zmynd10* CRISPR/Cas9 targeted larvae displaying severe body curvatures at 3 and 5 dpf did not survive to adulthood; however, we did observe that some F0 adult zebrafish displaying adult-viable scoliosis survived (Figure 4C). Whether this shift in phenotype is due to genetic chimerism and/or from the generation of hypomorphic alleles in a subset of injected zebrafish remains to be determined. To verify the efficiency of CRISPR/Cas9 system in this screening approach, we performed RT-PCR to determine *dnaaf1* or *zmynd10* mRNA expression in *dnaaf1* or *zmynd10* knockout zebrafish embryos with curvature phenotypes at 3 dpf. The results revealed ∼6.2-fold reduction in *dnaaf1* expression of curved *dnaaf1* CRISPR/Cas9 targeted larvae, as well as ∼4.6-fold decrease in *zmynd10* expression of curved *zmynd10* CRISPR/Cas9 targeted larvae (Supplementary Figure S5). In total, our multiplexed CRISPR/Cas9-based reverse genetic screen and analysis of zebrafish out to 30 dpf demonstrates that *dnaaf1*, *zmynd10*, and *tns1a* can generate mild to severe body axis curvatures in larval zebrafish (Figures 4A,B). Furthermore, targeting mutations against *zmynd10* and *dnaaf1* resulted in adult-viable F0 scoliosis mutant zebrafish, without obvious patterning defects of the vertebral column (Figure 4C), warranting further analysis of stable human AIS-associated alleles in zebrafish.

### Distribution Profiles of the Variants Relevant to Clinical Characteristics

**FIGURE 5:**
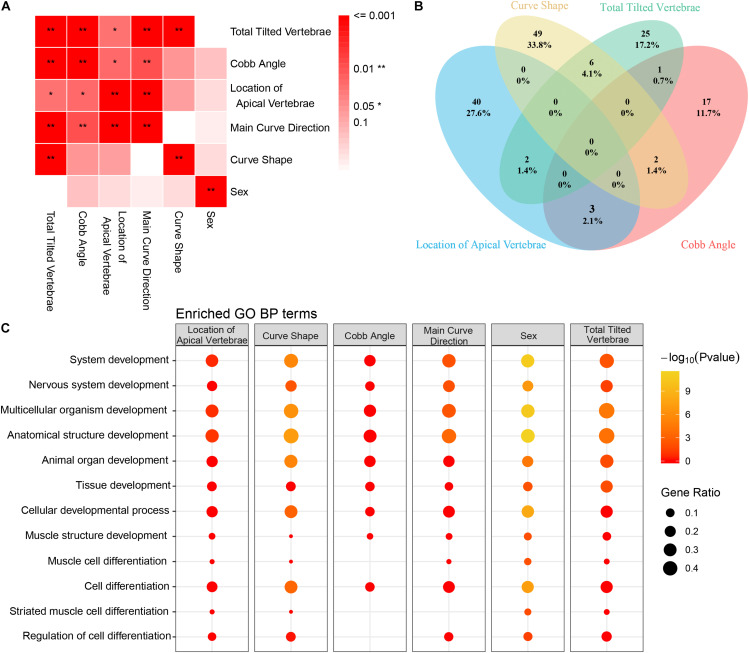
Correlation between clinical features and Gene Ontology (GO) enrichment analysis of clinical feature-related variants. **(A)** The association between each clinical feature is depicted. The strength of the association is represented by the intensity of the red color. **(B)** Venn diagram of the distribution of each clinical feature-related variant as identified in our study (blue circle = location of apical vertebrae, yellow circle = Cobb angle, green circle = main curve direction, and pink circle = number of tilted vertebrae). **(C)** GO enrichment analysis of the filtered variants was performed. GO biological process (BP) annotation is indicated on the left. Each individual box represents a clinical characteristic. The –Log10 (*P*-value) value is represented by color intensity, and the gene ratio is indicated by the size of the circle.

To determine if the variants we identified were associated with specific clinical manifestations, the AIS cohort was classified into subgroups by several clinical variables (Figure 5A). We found several significant associations among the vertebra structural characteristics; for instance, the total tilted vertebrae were strongly correlated with Cobb angle, the location of the apical vertebra of the curve, and curve shape (Figure 5A), which means that mutual influence may exist on these four variables. To determine the clinical characteristics associated SNVs, genetic differences between subgroups were compared and analyzed. Variants associated with each clinical characteristic are identified (Supplementary Table S5). The number of variants that were commonly or uniquely associated with those clinical variables was demonstrated by a Venn diagram (Figure 5B). The result showed that only one (0.1%) variant was shared by all four clinical variables. It means that the severity of each clinical feature may be affected by a unique variant subset. Larger sample studies are needed to confirm the underlying genotype–phenotype association.

Gene Ontology enrichment analysis of the variants was performed to reveal the differential development-associated terms specified for each characteristic (Supplementary Table S6). The GO annotation of part of the BP results was shown (Figure 5C). For all the clinical characteristics, except for “sex,” there were several terms consistently associated with different clinical features. The terms of regulation of nervous system development and regulation of multicellular organismal development were only enriched within curve shape and total tilted vertebrae. Coincidently, curve shape was only associated with total tilted vertebrae among all these clinical characteristics (Figure 5A). These observations support that certain development-related terms are associated with some clinical characteristics. Also, the KEGG pathway enrichment analysis result was provided (Supplementary Table S6).

### Correlation Between Genes of Key Gene Families and Adolescent Idiopathic Scoliosis

**FIGURE 6:**
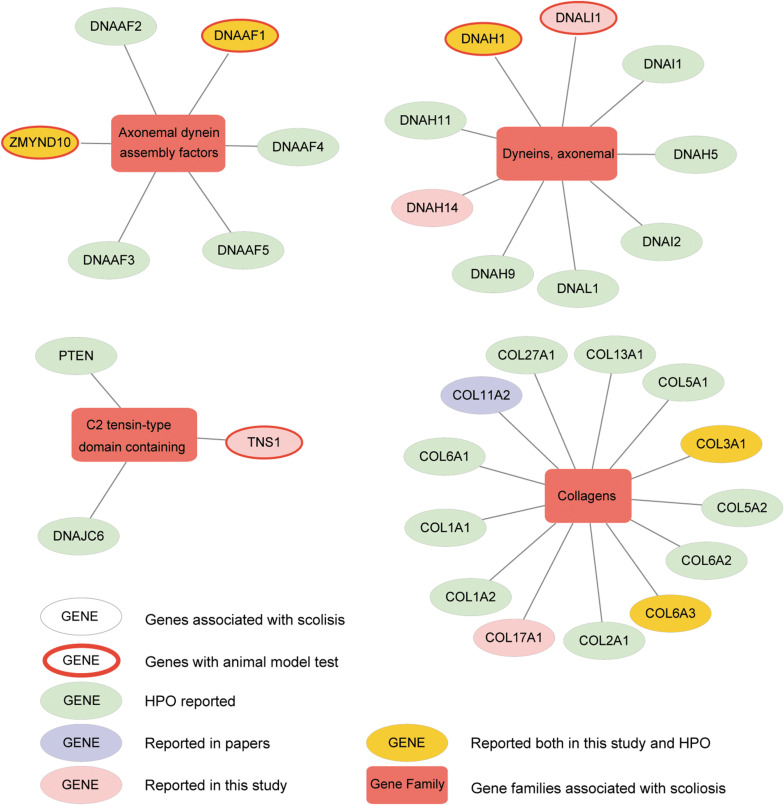
Altered genes and gene families in adolescent idiopathic scoliosis (AIS). The four gene families significantly enriched with mutated genes (Fisher exact test, adjusted *P*-value < 0.05) are shown. Circle means gene, and box means gene family associated with AIS. Genes with red ring are the ones identified to be associated with scoliosis in zebrafish after knockout. Genes in yellow circle are the ones reported both in our study and HPO (Human Phenotype Ontology). Genes in pink circle are the ones reported in our study. Genes in purple circle are the ones reported in published articles. Genes in light green circle are the ones reported in HPO.

To further understand the interactive relationship between the main gene families and AIS, the correlations reported in previous studies, Human Phenotype Ontology (HPO) database, and the current study were recorded. We presented four gene families significantly enriched with candidate genes from our analysis, including axonemal dynein assembly factors, dynein axonemal, C2 tensin-type domain containing, and collagens (Figure 6 and Supplementary Figure S6). We detected and verified five genes, DNAAF1, ZMYND10, DNALI1, DNAH1, and TNS1 of these gene families using bioinformatics analysis and *in vivo* knockout experiments. In a previous study, variants in collagen genes were significantly enriched in AIS patient samples (Haller et al., 2016). These AIS-associated gene families suggested several potential molecular regulatory mechanisms that may play roles in the development of AIS, including cytoskeletal change, regulation of actin cytoskeleton, extracellular matrix (ECM)–receptor interaction, and cell adhesion molecules. This is consistent with the current multi-hypothesis theories about the cause of AIS.

## Discussion

Early diagnosis and treatment of AIS in children are challenging due to a paucity of insight into the specific pathogenesis of the disease. Several theories have been proposed for the pathogenesis of AIS of which genetic factors are widely expected to play important roles. WES has been suggested to be an efficient way to identify pathogenic mutations in human complex diseases (Kiezun et al., 2012). With the falling cost burden of WES, and established public gene databases, it is possible to detect new susceptibility variants and genes by the case-only WES research design (Wu et al., 2015). Here in our study, WES and analysis were performed on a cohort of 195 AIS patient samples. All the AIS patients are sporadic in origin and from ethnic Han Chinese residing in Southern China. We focused on the analysis of 237 rare variants located in 232 genes. Subsequent analysis revealed that multiple gene families and pathways may be involved in the pathogenesis of disease and may drive isolated phenotypic characteristics of scoliosis. Using the zebrafish model, we tested a subset of five enriched genes and found that two of these gene targets lead to scoliosis, indicating new susceptibility loci for human AIS. At last, the gene families we identified highlighted several subsets of genes that might play a major role in the AIS pathogenesis. These potential genetic factors may broaden the comprehensive genetic delineation of AIS.

Ciliopathy-associated genes were studied and reviewed in AIS research, and many were found to be associated with scoliosis. WES of 73 AIS patient samples was performed to test for correlation between IS and elongated primary cilia (Oliazadeh et al., 2017); analysis showed an enrichment of variants in genes of cellular mechanotransduction, which was proposed to influence the function and structure of cilia. Additionally, rare variants of fibrillin-1 (FBN1) and fibrillin-2 (FBN2) and deleterious variants in ECM genes were found to be related to AIS (Buchan et al., 2014; Haller et al., 2016). In total, prior genetic studies of human AIS suggest a complex disease model. Therefore, high genetic heterogeneity has been implicated in these studies and is also supported by our result. In our enrichment analysis, dynein-associated gene families were detected, which means that these pathways were also involved in the pathogenesis of AIS of Chinese cohort. The dysfunction of cilia was also suggested to be related to AIS susceptibility in several other studies (Grimes et al., 2016; Oliazadeh et al., 2017; Konjikusic et al., 2018; Van Gennip et al., 2018; Zhang et al., 2018; Vesque et al., 2019). Mutations in *ptk7* or *kif6*, which affect the formation of ependymal cilia, induce IS-like scoliosis in zebrafish (Grimes et al., 2016; Konjikusic et al., 2018), implicating CSF flow as a crucial regulator of spine stability. Another important study suggested that loss of CSF flow leads to neuroinflammation, which was shown to be the target of treatment to ameliorate the onset and progression of scoliosis in zebrafish models of the disorder (Van Gennip et al., 2018).

Given that the dynein gene family in cilia development is one of the most enriched gene families in our current AIS cohort, the damaging effect of candidate variants in dynein genes *DNALI1*, *DNAH1*, *DNAAF1*, and *ZMYND10*, as well as one variant from fibrillin-related gene *TNS1*, were functionally tested in zebrafish. We conducted subsequent functional studies among those genes by using a multiplexed CRISPR/Cas9 targeted gene disruption approach because of its relatively high efficiency of assessing gene function. Targeted disruption of *dnaaf1* and *zmynd10* consistently generated adult-viable F0 mutant zebrafish with scoliosis. DNAAF1 is essential for the assembly of dynein heavy chain components and stability of the ciliary architecture (Loges et al., 2009). Mutations in *DNAAF1* are linked to tissue asymmetry development and primary ciliary dyskinesia (PCD) (Ferreira et al., 2018; Hartill et al., 2018). ZMYND10 is also required for the assembly of the dynein arms (Mali et al., 2018) and associated with PCD in humans (Moore et al., 2013). Loss of *zmynd10* in medaka fish led to viable scoliosis, with vertebral malformations (Kobayashi et al., 2017), further supporting our results and analysis of scoliosis phenotypes using chimeric F0 mutagenesis approach in zebrafish. Altogether, our data show that disruption of the cilia component encoding genes *dnaaf1* and *zmynd10* generates scoliosis in zebrafish. Furthermore, this warrants the targeted analysis of dynein-associated components in expanded AIS patient populations.

Several clinical features were analyzed in the present study (see section “Results”), and the Lenke classification was not adopted for subsequent analysis as it is a classification method to guide AIS treatment in humans. Five included clinical features were radiographic characteristics and partially following the suggestions of an annual meeting of the International Consortium for Spinal Genetics, Development, and Disease (ICSGDD) (Giampietro et al., 2018). The Cobb angle was the most commonly used clinical characteristic for assessing scoliosis severity. The significant differential variants between the larger and smaller Cobb angle groups determined in our study may contribute to curve progression. However, further studies of larger, independent cohorts are needed to verify this hypothesis. All the variants associated with specific clinical characteristics only have a small shared variant set. We therefore speculated that the gene sets of GO terms determined by enrichment analysis may contribute to specific clinical characteristics. Unexpectedly, those development-associated GO terms were commonly shared by all clinical characteristics, which means that no specific gene set is associated with particular clinical characteristics in the current results. Anyway, the results showed that the development-associated GO terms are correlated with isolated phenotypes of AIS. Sequencing studies with larger sample sizes are needed to reveal the underlying genotype–phenotype association.

In conclusion, our results presented a sporadic AIS study in the Southern Chinese population. We determined and verified a list of candidate genes that may correlate with AIS susceptibility and that several gene sets may correlate with AIS initiation or specific clinical phenotypes. The current study may contribute to the comprehensive depiction of genotype–phenotype association in AIS.

## Data Availability Statement

All data can be viewed in NODE (http://www.biosino.org/node) by pasting the accession (OEP000298) into the text search box or through the URL: http://www.biosino.org/node/project/detail/OEP000298.

## Ethics Statement

The studies involving human participants were reviewed and approved by the Ethics Committee of Xiangya Hospital of Central South University (Changsha, China). Written informed consent to participate in this study was provided by the participants’ legal guardian/next of kin. The animal study was reviewed and approved by the Animal Studies Committee at the University of Texas at Austin (AUP-2018-00342, study of spine development and disease using the zebrafish model).

## Author Contributions

YW collected patient samples and performed *in vivo* experiments. ZL organized and analyzed the WES data. GY and LiX collected and organized patient information. QG and CG set up the including criterion and screened AIS patients. JL extracted the DNA samples. BT and RG designed and supervised the zebrafish experiments. LuX designed and supervised the bioinformatics analysis. YW wrote the manuscript. RG and LuX revised the manuscript. HZ conceived and funded the project. All authors contributed to the article and approved the submitted version.

## Conflict of Interest

The authors declare that the research was conducted in the absence of any commercial or financial relationships that could be construed as a potential conflict of interest.

## Footnotes

1 https://software.broadinstitute.org/gatk/best-practices/

## Supplementary Material

Supplementary Figure 1 Genetic alteration in putative AIS-related genes (ZMYND10). **(A)** Lollipop plot for mapping mutations on the linear protein and its domains. L39P is the significant amino acid change caused by SNV on ZMYND10. **(B)** The detailed template information and models. The top template was selected and analyzed in the downstream analyses. **(C)** The sequence view displays the predicted secondary structure, the confidence in this prediction, the secondary structure of the model, the sequence and the modelled regions. The corresponding result scores for model assessment are shown with colors. **(D)** The sequence profile graph represents residue preferences at particular sequence position. **(E)** The predicted 3D structure of the mapping domain on ZMYND10.

Supplementary Figure 2 Genetic alteration in putative AIS-related genes (DNALI1). **(A)** Lollipop plot for mapping mutations on the linear protein and its domains. R135W is the significant amino acid change caused by SNV on DNALI1. **(B)** The detailed template information and models. The top template was selected and analyzed in the downstream analyses. **(C)** The sequence view displays the predicted secondary structure, the confidence in this prediction, the secondary structure of the model, the sequence and the modelled regions. The corresponding result scores for model assessment are shown with colors. **(D)** The sequence profile graph represents residue preferences at particular sequence position.

Supplementary Figure 3 Genetic alteration in putative AIS-related genes (DNAH1). **(A)** Lollipop plot for mapping mutations on the linear protein and its domains. P2228S is the significant amino acid change caused by SNV on DNAH1. **(B)** The detailed template information and models. The top template was selected and analyzed in the downstream analyses. **(C)** The sequence view displays the predicted secondary structure, the confidence in this prediction, the secondary structure of the model, the sequence and the modelled regions. The corresponding result scores for model assessment are shown with colors. **(D)** The sequence profile graph represents residue preferences at particular sequence position.

Supplementary Figure 4 Genetic alteration in putative AIS-related genes (TNS1). **(A)** Lollipop plot for mapping mutations on the linear protein and its domains. H608Y is the significant amino acid change caused by SNV on TNS1. **(B)** The detailed template information and models. The top template was selected and analyzed in the downstream analyses. **(C)** The sequence view displays the predicted secondary structure, the confidence in this prediction, the secondary structure of the model, the sequence and the modelled regions. The corresponding result scores for model assessment are shown with colors. **(D)** The sequence profile graph represents residue preferences at particular sequence position.

Supplementary Figure 5 Gene expression of *dnaaf1* and *zmynd10* knock-out zebrafish. **(A,B)** Quantitative real-time PCR analysis of *dnaaf1* and *zmynd10* expression in *dnaaf1* and *zmynd10* knock-out zebrafish. **p* < 0.05 versus the WT group (*n* = 3, Bars represent mean ± SD). WT, wild-type; KO, knock-out.

Supplementary Figure 6 Altered genes and gene families in AIS. The AIS associated gene families with mutated genes reported in this study are shown. Circle means gene and box means gene family associated with AIS. Genes with red ring are the ones identified associated with scoliosis in zebrafish after knockout. Genes in yellow circle are the ones reported both in our study and HPO. Genes in pink circle are the ones reported in our study. Genes in purple circle are the ones reported in published papers. Genes in light green circle are the ones reported in HPO (human phenotype ontology).

Supplementary Table 1 The clinical characteristics of the 195 included patients.

Supplementary Table 2 Summary of the 237 candidate variants.

Supplementary Table 3 The list of gene families involved in the pathophysiological changes of AIS.

Supplementary Table 4 The results of GSEA, gProfileR and GO analyses. KEGG pathway enrichment analysis by GSEA; GO enrichment analysis by GSEA; gProfileR analysis.

Supplementary Table 5 Summary of the variants identified to be related to clinical variables. Including the variants identified to be related to Cobb angle, the variants identified to be related to curve shape, the variants identified to be related to the location of apical vertebrae, the variants identified to be related to main curve direction, the variants identified to be related to sex and the variants identified to be related to the total tilted vertebrae.

Supplementary Table 6 GO and KEGG pathway enrichment analysis of the clinical characteristic-associated variants, refer to Cobb angle, curve shape, the location of apical vertebrae, main curve direction, sex, and the total tilted vertebrae, respectively.

Supplementary Table 7 The oligo sequences for all the injections.

Supplementary Table 8 The zebrafish counting of post injection for each gene.
