# Supplementary material for: Coding Variants Coupled With Rapid Modeling in Zebrafish Implicate Dynein Genes, dnaaf1 and zmynd10, as Adolescent Idiopathic Scoliosis Candidate Genes
Source: Front Cell Dev Biol. 2020 Nov 4;8:582255. doi: 10.3389/fcell.2020.582255 (PMC7672046; doi:10.3389/fcell.2020.582255)
Supplement: Supplementary Figure 1 — Genetic alteration in putative AIS-related genes (ZMYND10). (A) Lollipop plot for mapping mutations on the linear protein and its domains. L39P is the significant amino acid change caused by SNV on ZMYND10. (B) The detailed template information and models. The top template was selected and analyzed in the downstream analyses. (C) The sequence view displays the predicted secondary structure, the confidence in this prediction, the secondary structure of the model, the sequence and the modelled regions. The corresponding result scores for model assessment are shown with colors. (D) The sequence profile graph represents residue preferences at particular sequence position. (E) The predicted 3D structure of the mapping domain on ZMYND10. [file Data_Sheet_2.docx]

Coding variants coupled with rapid modeling in zebrafish implicate dynein genes, dnaaf1 and zmynd10, as adolescent idiopathic scoliosis candidate genes

Yunjia Wang^1,2,3^, Zhenhao Liu^4,5^, Guanteng Yang^1,2^, Qile Gao^1,2^, Lige Xiao^1,2^, Jiong Li^1,2^, Chaofeng Guo^1,2^, Benjamin R. Troutwine^3^, Ryan S. Gray^3^, Lu Xie^4^, Hongqi Zhang^1,2^*

Supplementary Material

**Supplemental table S1.** The clinical characteristics of the 195 included patients.
**Supplemental table S2.** Summary of the 237 candidate variants.
**Supplemental table S3.** The list of gene families involved in the pathophysiological
changes of AIS.
**Supplemental table S4.** The results of GSEA, gProfileR and GO analyses. KEGG
pathway enrichment analysis by GSEA; GO enrichment analysis by GSEA; gProfileR
analysis.

**Supplemental table S5.** Summary of the variants identified to be related to clinical
variables. Including the variants identified to be related to Cobb angle, the variants
identified to be related to curve shape, the variants identified to be related to the
location of apical vertebrae, the variants identified to be related to main curve
direction, the variants identified to be related to sex and the variants identified to be
related to the total tilted vertebrae.

**Supplemental table S6.** GO and KEGG pathway enrichment analysis of the clinical characteristic-associated variants, refer to Cobb angle, curve shape, the location of apical vertebrae, main curve direction, sex and the total tilted vertebrae, respectively.

**Supplemental table S7.** The oligo sequences for all the injections.
**Supplemental table S8.** The zebrafish counting of post injection for each gene.
**Supplemental figures S1.** Genetic alteration in putative AIS-related genes (ZMYND10).

**Supplemental figures S2.** Genetic alteration in putative AIS-related genes (DNALI1).

**Supplemental figures S3.** Genetic alteration in putative AIS-related genes (DNAH1).

**Supplemental figures S4.** Genetic alteration in putative AIS-related genes (TNS1).

**Supplemental figures S5.** Gene expression of *dnaaf1* and *zmynd10* knock-out zebrafish.

**Supplemental figures S6.** Altered genes and gene families in AIS.

**
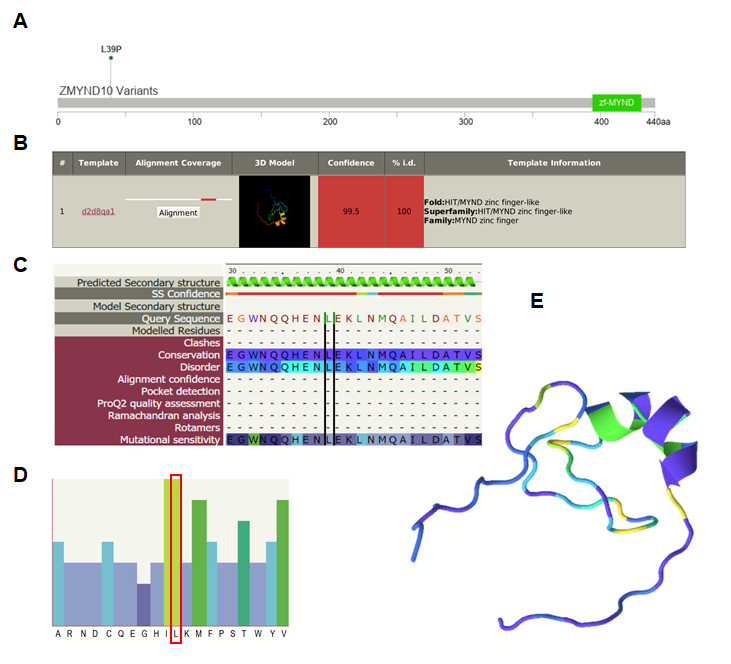
**

**Supplementary Figure 1. Genetic alteration in putative AIS-related genes (ZMYND10).**

(A) Lollipop plot for mapping mutations on the linear protein and its domains. L39P is the significant amino acid change caused by SNV on ZMYND10. (B) The detailed template information and models. The top template was selected and analyzed in the downstream analyses. (C) The sequence view displays the predicted secondary structure, the confidence in this prediction, the secondary structure of the model, the sequence and the modelled regions. The corresponding result scores for model assessment are shown with colors. (D) The sequence profile graph represents residue preferences at particular sequence position. (E) The predicted 3D structure of the mapping domain on ZMYND10.

**
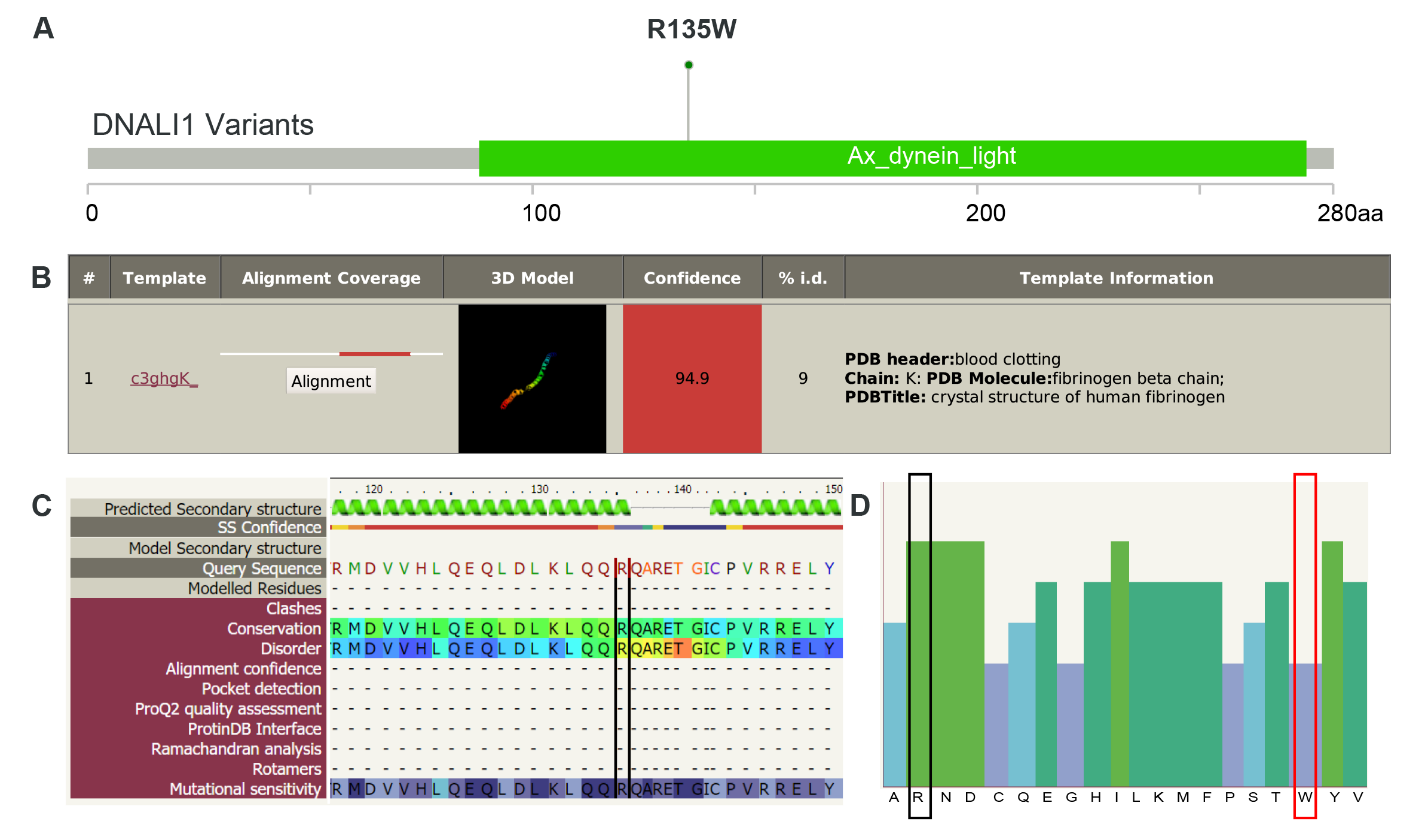
**

**Supplementary Figure 2. Genetic alteration in putative AIS-related genes (DNALI1).**

(A) Lollipop plot for mapping mutations on the linear protein and its domains. R135W is the significant amino acid change caused by SNV on DNALI1. (B) The detailed template information and models. The top template was selected and analyzed in the downstream analyses. (C) The sequence view displays the predicted secondary structure, the confidence in this prediction, the secondary structure of the model, the sequence and the modelled regions. The corresponding result scores for model assessment are shown with colors. (D) The sequence profile graph represents residue preferences at particular sequence position.


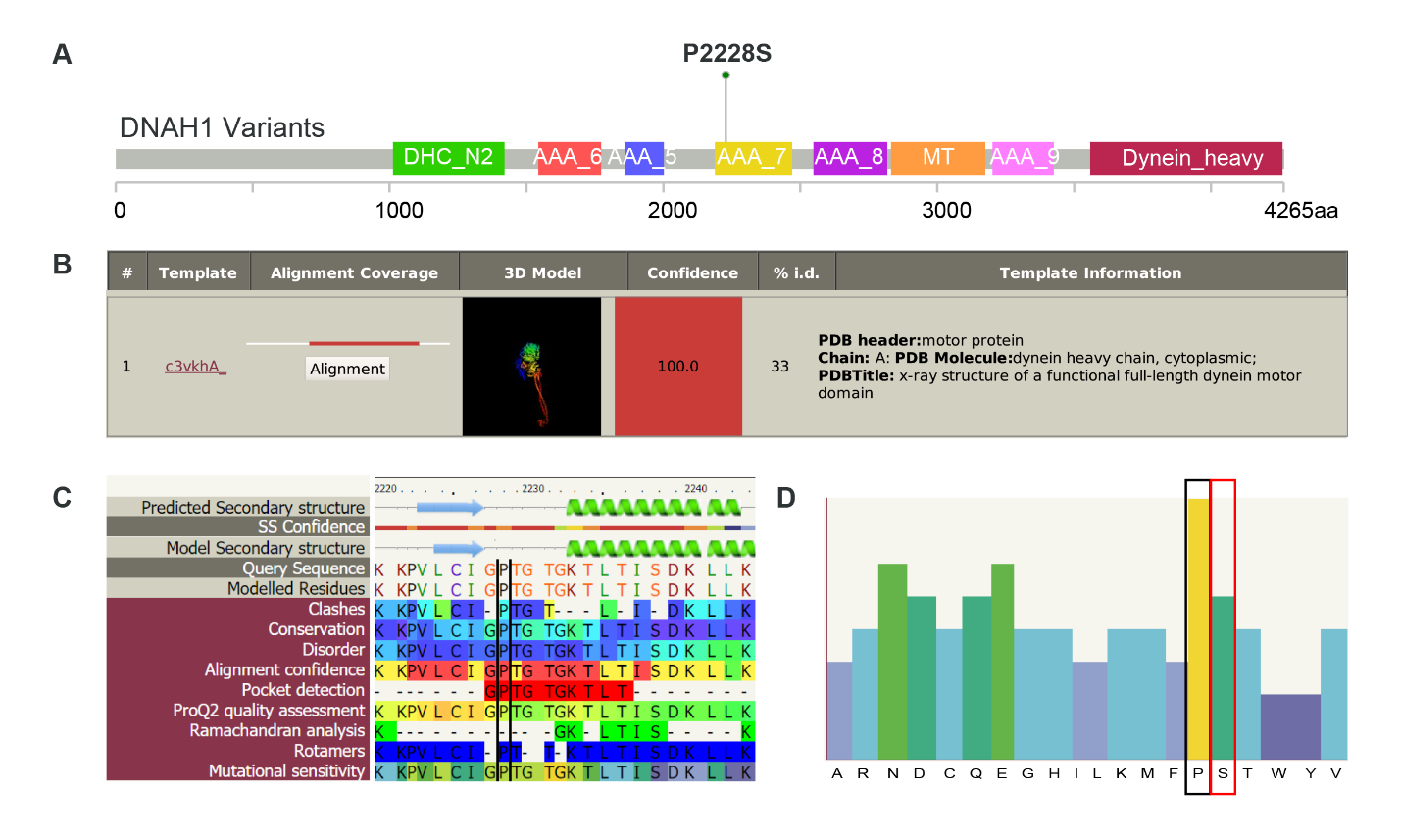


**Supplementary Figure 3. Genetic alteration in putative AIS-related genes (DNAH1).**

(A) Lollipop plot for mapping mutations on the linear protein and its domains. P2228S is the significant amino acid change caused by SNV on DNAH1. (B) The detailed template information and models. The top template was selected and analyzed in the downstream analyses. (C) The sequence view displays the predicted secondary structure, the confidence in this prediction, the secondary structure of the model, the sequence and the modelled regions. The corresponding result scores for model assessment are shown with colors. (D) The sequence profile graph represents residue preferences at particular sequence position.


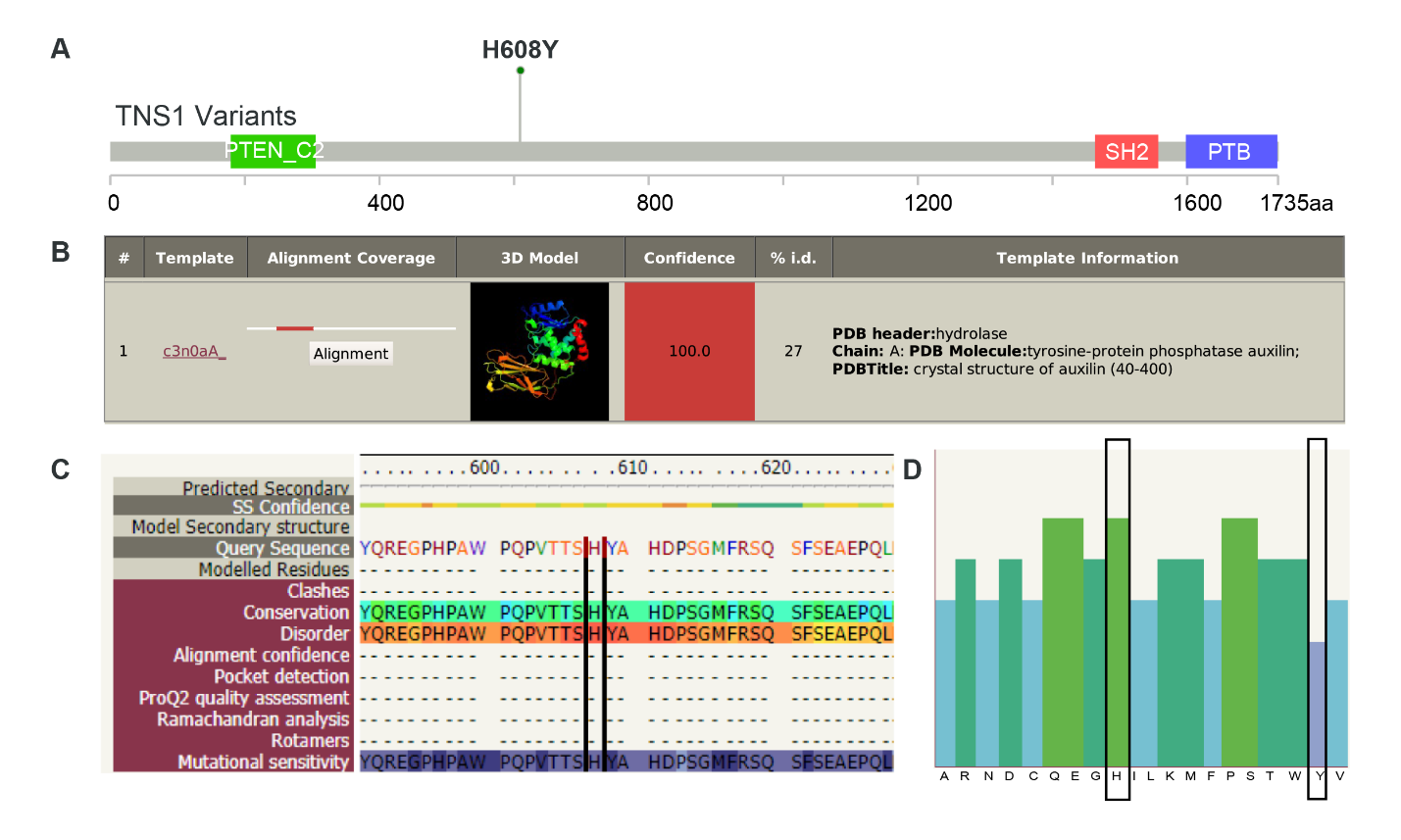


**Supplementary Figure 4. Genetic alteration in putative AIS-related genes (TNS1).**

(A) Lollipop plot for mapping mutations on the linear protein and its domains. H608Y is the significant amino acid change caused by SNV on TNS1. (B) The detailed template information and models. The top template was selected and analyzed in the downstream analyses. (C) The sequence view displays the predicted secondary structure, the confidence in this prediction, the secondary structure of the model, the sequence and the modelled regions. The corresponding result scores for model assessment are shown with colors. (D) The sequence profile graph represents residue preferences at particular sequence position.


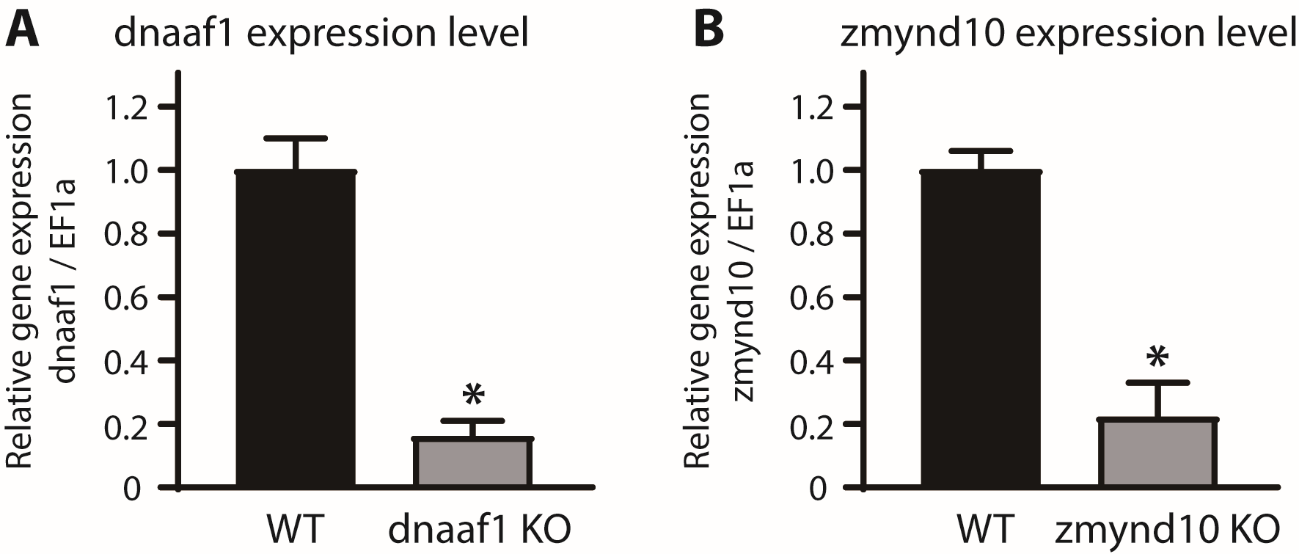


**Supplemental figures 5. Gene expression of *dnaaf1* and *zmynd10* knock-out zebrafish.**

(A and B) Quantitative real-time PCR analysis of *dnaaf1* and *zmynd10* expression in *dnaaf1* and *zmynd10* knock-out zebrafish. * *p* < 0.05 versus the WT group (n = 3, Bars represent mean ± SD). WT, wild-type; KO, knock-ou
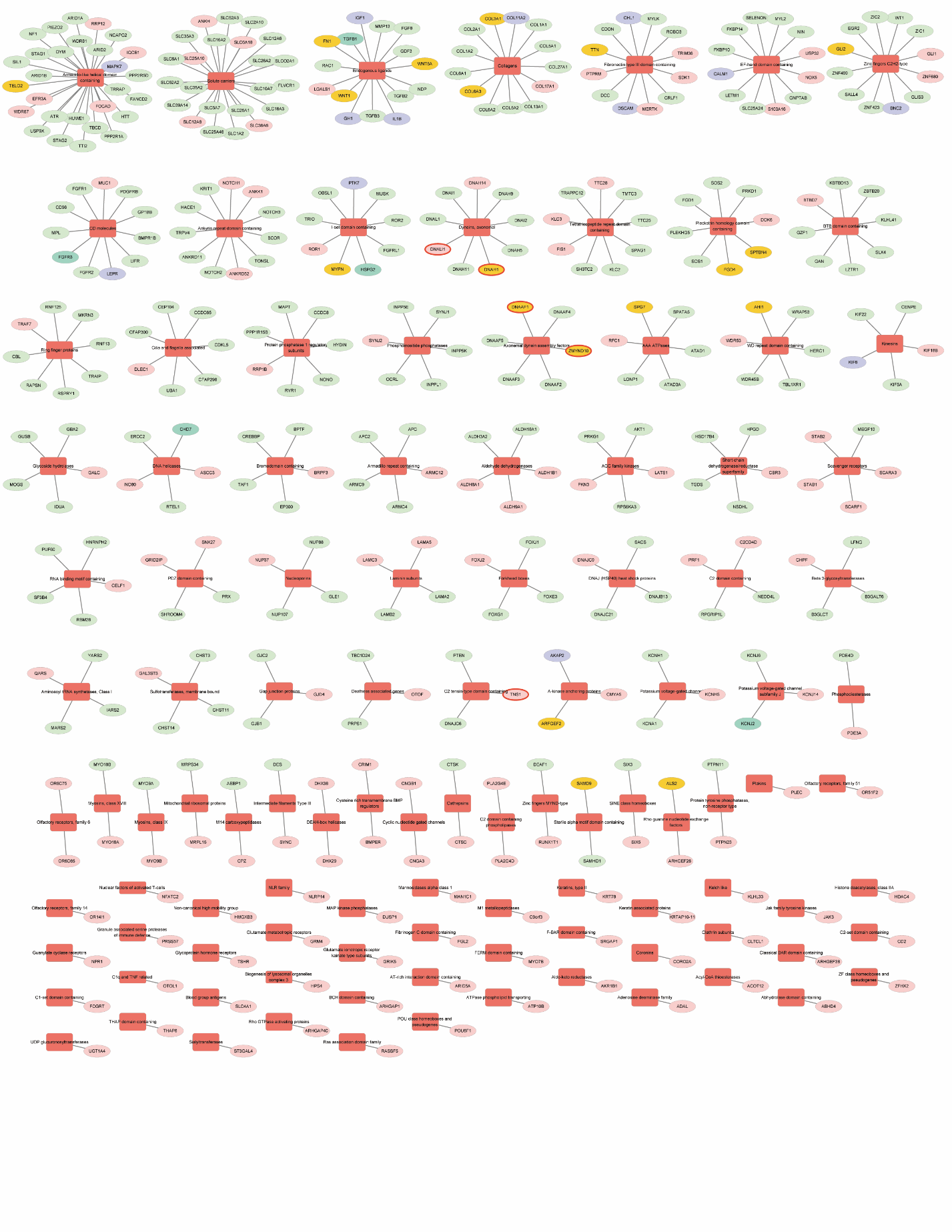


**Supplementary Figure 6. Altered genes and gene families in AIS**

The AIS associated gene families with mutated genes reported in this study are shown. Circle means gene and box means gene family associated with AIS. Genes with red ring are the ones identified associated with scoliosis in zebrafish after knockout. Genes in yellow circle are the ones reported both in our study and HPO. Genes in pink circle are the ones reported in our study. Genes in purple circle are the ones reported in published papers. Genes in light green circle are the ones reported in HPO (human phenotype ontology).
